# Editorial: Creativity, innovation, and entrepreneurship: The learning science toward higher order abilities

**DOI:** 10.3389/fpsyg.2022.1063370

**Published:** 2022-11-29

**Authors:** Zehui Zhan, Patrick S. W. Fong, Kuen-Yi Lin, Baichang Zhong, Harrison Hao Yang

**Affiliations:** ^1^School of Information Technology in Education, South China Normal University, Guangzhou, China; ^2^Department of Building and Real Estate, Hong Kong Polytechnic University, Kowloon, Hong Kong SAR, China; ^3^Department of Technology Application and Human Resource Development, Institute for Research Excellence in Learning Sciences, National Taiwan Normal University, Taipei, Taiwan; ^4^School of Education, State University of New York at Oswego, Oswego, NY, United States

**Keywords:** creativity, innovation, entrepreneurship, learning science, higher order abilities

Creativity, innovation, and entrepreneurship are emphasized as the major power in driving the development of our fast-changing world. Especially in the era of Industry 4.0, where intelligent manufacturing plays an important role, creative and entrepreneurial talents have gained more attention than ever before. Cultivating and educating those talents has become a key issue that needs to be solved. However, creativity, innovation, and entrepreneurship, as higher-order abilities of talents, are not achieved overnight, because they may be affected by a variety of complex factors, including innate and acquired. Available knowledge about the teaching and learning of students' higher order abilities is still insufficient and the challenges of linking theories and practices claim more research efforts into mature pedagogies, effective teaching aids, and accurate evaluation tools. Hence, the present Research Topic on “Creativity, innovation, and entrepreneurship: The learning science toward higher order abilities” contributes with updates and different perspectives on innovation-oriented education, representing the effort of 29 academic papers from 91 authors in total. The following section will elaborate contributions of these papers to the Research Topic.

## Creativity and innovation

Creativity is regarded as the fountainhead of human civilizations, different strategies, and technologies were introduced to facilitate it.

Game playing is seen as a potential way to engender greater fun and thus more creativity may be facilitated. Mun conducted two experiments on university students to explore the effect of game playing and goal orientation on creativity. Results showed that the cognitive game that engendered the greatest degree of fun led to more creative outcomes. In Experiment 1, the cognitive game that engendered greater degree of fun resulted in more creative outcomes on a subsequent new product development task, compared with the control group and the cognitive game that did not engender as much fun. Results of Experiment 2 showed the effects of goal orientations on creative outcomes (i.e., focus on the process of playing the game vs. focus on the outcome of winning) in terms of novelty, usefulness, and overall creativity. It confirmed that individuals who participated in the process of goal-oriented cognitive game that engendered a high degree of fun were more creative on the subsequent toy design task than those who were given an outcome goal orientation.

Zhan et al. explored the effectiveness of product-based pedagogy (PBP) on high school students' creativity and innovative thinking in an artificial intelligence course. After employing a seven-step teaching model (i.e., phenomenon, problem, plan, prototype, product, presentation, price) in accordance with PBP, in which the key function of the product as a linkage between creativity and innovation was emphasized, positive results were found in the treatment group students' project management skills, creativity, and innovative thinking. In future, more AI curriculum are expected to be developed for students to participate easily in the process of product creation, and learn more effectively to develop creativity and innovative thinking.

To cultivate senior high school students' creativity, Wang et al. conducted a study about maker teaching activity design in general technology course. By three rounds of action research, a teaching model was proposed to improve students' creativity effectively, which synthesized the Four Periods of Creative Process (i.e., preparation, gestation, enlightenment and verification) and the Five Stages of Creative Problem Solving (i.e., discovering facts, discovering problems, seeking ideas, seeking solutions and seeking acceptance). Finally, according to the comparative experimental study, it was found that the teaching model could improve students' creativity significantly and effectively. Significant positive improvement was also found on students' adventurous, curiosity, imagination and challenge.

Yan et al. addressed that it was a worthy topic on developing sustainable interventions to promote students' self-efficacy in creativity without generating excessive workload for teachers. In their study, the self-assessment mind maps were employed as instructional intervention, and their effects on students' self-efficacy in creativity, self-efficacy in learning English, and academic performance in English language tests were examined. Empirical study was conducted in a Hong Kong primary school. Results showed that, after the intervention, while students' self-efficacy and test performance in English learning were not improved, there was significant positive effect on self-efficacy in creativity.

Fan and Ye focused on the application of inquiry-based teaching and learning in project design courses at university level in Taiwan. Quasi-experimental design method was adopted to examine the effect of two inquiry models. Results of five questionnaire surveys during the design project process showed positive effect on students' curriculum interest, curriculum value perception, and curriculum confidence.

Zhang et al. conducted a review on problem-based learning (PBL) research conducted over the past 40 years (from 1981 to 2021). They analyzed a total of 2,790 articles and reviews, and concluded that current research hotspots focus on the extensions of PBL teaching mode, application of PBL teaching method, and reform of PBL. Major contributors, key researchers and publishers were also listed. Overall, research on PBL has continued to increase over the past few decades. The authors highlighted that setting the right questions are the core of PBL, setting up the curriculum, and designing the questions according to the learning objectives are the key issues in PBL.

According to Chen et al., while students' creative behaviors were not significantly improved, the argument map (AM)-supported online group debate activities were helpful for college students' critical thinking, including their depth and phases of critical thinking. They also emphasized on teachers' real-time feedback for students' improvements of high-level thinking skills and progress of the activity.

Referring to the theory of planned behavior, Tzeng et al. adopted self-evaluation as an intermediate variable to predict college students' adoption of technology for self-directed learning. In total of 285 college students participated in their survey, and the authors found that self-evaluation enhanced the influence of intentions on behavior and improved the accuracy of predictions of college students' adoption of technology for self-directed learning. They highlight the importance of students' attitudes and perceived behavioral control on intention.

Based on self-determination theory, Han et al. proposed that psychological safety positively affects students' creativity through psychological empowerment, and fault-tolerant culture also played a positive role in it. They conducted questionnaire survey on 238 students in China, and confirmed a positive correlation between psychological safety and creativity. The mediating role of psychological empowerment in the relationship between them was also revealed. Moreover, results showed that a fault-tolerant culture played a moderating role between psychological safety and psychological empowerment. Specifically, the fault-tolerant culture enhanced the direct influence of psychological safety on psychological empowerment and the indirect influence of psychological safety on creativity.

Creative role identity is also seen as an important antecedent for the encouragement of individuals to produce innovative behaviors. Deng et al. analyzed the psychological mechanism (flow as a mediator) of the influence of innovation climate on individual creative role identity of university students. Results of the questionnaire survey data collected from 226 students confirmed that an innovation climate has a significant positive impact on the identity of individual creative roles, and flow mediated the relationship between innovation climate and creative role identity.

Cui et al. formed a research framework to explore the correlations among hands-on making attitude, Interest type Epistemic Curiosity (IEC) and Deprived type Epistemic Curiosity (DEC), and career interest. They collected data from 220 participants in the 2021 Taiwan International Exhibition of Young Inventors (IEYI), in which young students were encouraged to make innovative projects by applying STEM (science, technology, engineering, and mathematics) knowledge and collaborative design. Results showed positive correlations between hands-on making attitude and the two types of epistemic curiosity. There were also positive correlations between STEM career interest and the two types of epistemic curiosity, and DEC had a higher coefficient on STEM career interest than IEC. In addition, both types of epistemic curiosity had a mediating role between hands-on making attitude and STEM career interest.

Jónsdóttir and Macdonald introduced a model that could help teachers to identify and analyze their teaching and learning process to support students' creativity at any school level and in any subject. The model was found based on the sociologist, Basil Bernstein's concepts, and drew on sociological concepts such as “framing and classification” and “power and control” in school settings.

Combining microcomputer interfaces and network communication technologies as well as virtual instrumentation, Wang designed an internet-based psychophysiological response testing and analysis system for creative learning. Differed from questionnaires and self-assessments, the system could be used to collect information on learners' psychophysiological responses and real-time performance of student participants during creative learning; especially when stress might affect creativity, the system was designed to investigate the relationship between different stress levels and performance on different creative learning tasks.

In addition to individuals' creativity, team creativity was also investigated in this Special Issue. To investigate the impact of leadership behavior to team creativity in startup teams, Antonio et al. proposed two mediator variables: Team Climate and Team Ambidexterity, and conducted an empirical quantitative research with more than 434 participants, aggregated into 145 teams. Samples are early startup teams in several cities in Indonesia, ran and led by young people. The result showed that Team Climate and Team Ambidexterity are good mediators of Servant and Transformational leadership behaviors to Team Creativity in startup teams. Kim and Kim discussed the public innovation capacity in Korea. They topologized public innovation capacity in terms of individuals, middle managers, and organizations' levels through mini-round Delphi analysis. They validated public innovation capacity through a questionnaire survey of 477 public employees from 30 agencies.

Considering that creativity does not always lead to positive outcomes, Dou et al. conducted a qualitative comparative analysis of negative and malevolent creativity. They concluded that negative creative thinking is a kind of native thinking based on personal interests that are developed to emphasize the benefits of an individual's interests, while malevolent creative thinking is a kind of native thinking based on the value-added of personal interests and is deliberately harmful. Identify the similarities (e.g., share a value orientation, environmental stimulation, and subjective motivation) and differences (e.g., differ in terms of value goals, ways of thinking, and the scale of the subject) in connotations among them, they proposed the Negative-Malevolent Thinking Interconnection Model (NMTIM), a linkage model of negative creative thinking and malevolent creative thinking to better show the bidirectional linkage mechanism.

## Entrepreneurship

Exploring the factors influencing entrepreneurial intention is crucial to entrepreneurial practices and education.

Luo et al. studied the mediation effects of social capital and human capital on the relationship between proactive personality and entrepreneurial intentions in college students. After testing a sample of 300 Chinese college students, results showed that college students' proactive personality exerted a significant and positive impact on their entrepreneurial intentions. Social capital and human capital both played a partial mediating role between the proactive personality and entrepreneurial intentions. In addition, the study further discovered the chain mediating role of social capital and human capital between proactive personality and entrepreneurial intention. It was believed that Chinese college students' social capital will significantly influence their human capital. In other words, college students with a higher level of proactive personality will have more social capital and human capital, facilitating their generation of entrepreneurial intentions.

Considering personal values in an entrepreneurial process, Li et al. explored the relationship between materialism and college students' entrepreneurial intention through a serial mediation model. In total of 1,002 Chinese university students participated in the online survey and completed the measurement of entrepreneurial intention, entrepreneurial attitude, materialism, and achievement motivation. The study found that materialism positively predicted college students' entrepreneurial intention, and this relationship was serially mediated through achievement motivation and entrepreneurial attitude.

Mei et al. studied the mediating role of commitment and moderating role of family support on university students' successive development from entrepreneurial intention to behavior. In total of 469 valid responses were obtained by a survey conducted among university students from six major universities in South China. Results showed both direct and indirect positive effects between entrepreneurial intention and entrepreneurial behavior, while entrepreneurial commitment and family support played mediating and moderating role between them respectively. It was concluded that entrepreneurial commitment bridged the path from entrepreneurial intention to behavior, and family support created the boundary effect.

Wang and Huang analyzed the mediating role of entrepreneurial self-efficacy and prosocial tendency in the relation between college students' post-traumatic growth and entrepreneurial intention in the post-COVID-19 era. They collected data from 690 Chinese undergraduates, and concluded that in the post-COVID-19 era, the post-traumatic growth of college students would have a significant and positive effect on their entrepreneurial intentions. Besides, results indicated the mediation role of students' entrepreneurial self-efficacy and prosocial tendencies between post-traumatic growth and entrepreneurial intentions, and the chain mediating effect between students' entrepreneurial self-efficacy and prosocial tendencies was also established.

Li et al. focused the effect of university entrepreneurship education on independent student entrepreneurship. They discussed the correlation between three factors (i.e., entrepreneurship education, entrepreneurial opportunity identification, entrepreneurial experience) and independent entrepreneurship. The authors collected questionnaire survey data from 1,424 fresh graduates who have received entrepreneurship education in China. Results showed that entrepreneurship theory-based courses could promote independent entrepreneurship, but entrepreneurship practice training surprisingly failed to promote. It reflected that graduates who have received entrepreneurial practice training might be more objective to evaluate the risks and difficulties of entrepreneurship, leading to more cautious considerations in choosing independent entrepreneurship. They also indicated that entrepreneurial opportunity identification mediated only between theory-based courses and independent entrepreneurship.

Li et al. explored the mediating role of creativity on the relationship between personality traits and entrepreneurial intention. In total of 674 valid questionnaires were collected from college students in China. Results showed that neuroticism in personality traits had significant negative impact on entrepreneurial intention and creativity, while conscientiousness, openness, and extraversion had significant positive impact. Creativity had significant positive impact on entrepreneurial intention, it had partial mediating role between neuroticism, conscientiousness, extraversion, and entrepreneurial intention along with complete mediating role between openness and entrepreneurial intention. Gao and Huang studied the mediation role of entrepreneurial self-efficacy between narcissistic personality and entrepreneurial intention. By conducting questionnaire survey sampled from 252 vocational college students in China, they found that narcissistic personality had a significant positive effect on entrepreneurial intention and entrepreneurial self-efficacy. Entrepreneurial self-efficacy had a significant positive effect on entrepreneurial intention and played a partial mediation role in the relationship between narcissistic personality and entrepreneurial intention.

Shadiev et al. studied students' creativity, innovation, and entrepreneurship in a telecollaborative project. Participants were at different age levels; one group was junior high school students from China (*n* = 15) and another group was university students from Indonesia (*n* = 10). Supported by the 360-degree video technology, students created cultural learning content and communicated with their international partners through a telecollaborative platform. Results showed that participants' creativity, innovation, and entrepreneurship were improved, and positive learning experiences were perceived.

Peng et al. studied the impact of resource bricolage on entrepreneurial orientation in startups. The moderating roles of top management team (TMT) heterogeneity and TMT behavioral integration were tested from the data of 295 startups. Results showed that the entrepreneurial orientation was positively correlated with the strategy of resource bricolage, and the relationship is positively moderated by TMT heterogeneity, while negatively moderated by TMT behavioral integration.

Yu et al. discussed the influence of knowledge management process and intellectual capital on innovation with the mediating effect of entrepreneurial orientation and moderating role of leaders' education levels. Based on the data of 393 IT firms in Pakistan, they discovered that innovation was positively correlated with knowledge management process and intellectual capital, and confirmed that entrepreneurial orientation partially mediated the relationship between knowledge management and intellectual capital on innovation. Moreover, it was confirmed that the moderation effect of leaders' education on intellectual capital and innovation relationship was insignificant.

Considering the gap in knowledge sharing in information system integration service industry, Hong et al. developed a research model to explore the mediating role of four types of knowledge sharing (i.e., automatic response, rational reflection, ridiculed reflection, and stolen reflection) in the relationship between problem solving self-efficacy (PSSE) and IT workers' job performance. Questionnaire results from 307 system integration IT workers showed that PSSE could positively predict four knowledge sharing types. Except for stolen reflection, job performance was positively predicted by the other three knowledge sharing types. It was concluded that supported by PSSE, job performance could be enhanced by automatic response systems and rational reflection systems in knowledge sharing.

New product development is a creative activity that requires to empathize users' needs. To this end, Chen et al. explored the impact of scenarios on the performance of entrepreneurial imaginativeness. Results confirmed that familiar scenarios that matched designers' background (including knowledge, expertise, and experience) could inspire entrepreneurial imaginativeness more than unfamiliar scenarios. They suggested that individuals utilize different familiar scenarios to foster their entrepreneurial imaginativeness. For team compositions, they proposed leaders to select members who were familiar with the task scenarios and had high entrepreneurial imaginativeness to ensure that the knowledge, expertise, and experience of the members could benefit the creative tasks.

Peljko and Antončič focused on entrepreneurial openness and creativity on entrepreneurial level relative to business growth. Structural equation modeling was employed to analyze survey data obtained from 851 entrepreneurs of small and medium-sized enterprises' (SMEs) in three countries. Results indicated that creativity of the entrepreneur was positively correlated with entrepreneurial openness and creative personality, and the growth of the firm was positively correlated with the entrepreneur's creativity. In addition to direct effects, some smaller indirect effects were detected in the model for the indirect effect of entrepreneurial openness and creative personality on growth through the creativity of the entrepreneur. The three cross-national comparative study contributes to comparative international entrepreneurship research.

Based on previous research results, we believe that these researches solve some important research gaps and have important contributions in the field of creativity, innovation, and entrepreneurship. However, some good research suggestions for further studies are also proposed in these researches. It is suggested that we could make more efforts based on these valuable research suggestions. Lastly, we extend deep gratitude to all authors and reviewers participated in this special issues. We value your articles and comments, and remain committed to striving for excellence in the future.

## Author contributions

All authors listed have made a substantial, direct, and intellectual contribution to the work and approved it for publication.

## Funding

This work was funded by the National Natural Science Foundation in China (62277018 and 62237001), Ministry of Education in China Project of Humanities and Social Sciences (22YJC880106), the Major Project of Social Science in South China Normal University (ZDPY2208), and the Major Basic Research and Applied Research Projects of Guangdong Education Department (#2017WZDXM004).

## Conflict of interest

The authors declare that the research was conducted in the absence of any commercial or financial relationships that could be construed as a potential conflict of interest.

## Publisher's note

All claims expressed in this article are solely those of the authors and do not necessarily represent those of their affiliated organizations, or those of the publisher, the editors and the reviewers. Any product that may be evaluated in this article, or claim that may be made by its manufacturer, is not guaranteed or endorsed by the publisher.

